# Drusen-specific dark adaptation profiles in intermediate age-related macular degeneration

**DOI:** 10.1186/s40942-025-00783-1

**Published:** 2026-02-07

**Authors:** Paolo Forte, Lorenzo Ferro Desideri, Marco Nassisi, Federica Milanesi, Alessandro Feo, Irene Bagnasco, Sharlot M. Iglesia, Giovanni Forte, Davide Scandella, Fabiola Roccatagliata, Vincenzo Fontana, Chiara Maria Eandi, Michele Iester, Jasleen K. Jolly, Martin S. Zinkernagel, Francesco Viola, Massimo Nicolò

**Affiliations:** 1 DINOGMI, University of Genoa, Genoa, Italy; 2 Eye Unit, IRCCS Ospedale Policlinico San Martino, Genoa, Italy; 3 Fondation Asile des Aveugles, Department of Ophthalmology, Jules-Gonin Eye Hospital, University of Lausanne, Lausanne, Switzerland; 4 Department of Ophtalmology, Inselspital, Bern University Hospital, Bern, Switzerland; 5 Bern Photographic Reading Center, Iselspital, University Hospital Bern, Bern, Switzerland; 6 Ophthalmology Unit, Fondazione IRCCS Ca’ Granda Ospedale Maggiore Policlinico, Milan, Italy; 7 Department of Clinical Sciences and Community Health, University of Milan, Milan, Italy; 8 Department of Biomedical Sciences, Humanitas University, Via Rita Levi Montalcini 4, 20072. Pieve Emanuele-Milan, Milan, Italy; 9 Department of Clinical-Surgical, Diagnostic and Pediatric Sciences, University of Pavia, Pavia, Italy; 10 ARTORG Research Center Biomedical Engineering Research, University of Bern, Bern, Switzerland; 11 RINA Consulting S.p.A, Genoa, Italy; 12 Department of Mathematics, University of Genoa, Genoa, Italy; 13 Department of Optometry and Vision Science, University of Melbourne, Melbourne, VIC, Australia; 14 Jolly Vision Science, Cambridge, UK

**Keywords:** Intermediate age-related macular degeneration, Drusen, AdaptDx, Rod-mediated dark adaptation, Dark adaptometry, Cuticular drusen, Soft drusen, Reticular pseudodrusen, iAMD

## Abstract

**Aims:**

To investigate the influence of macular drusen phenotypes on dark adaptation (DA) in intermediate age-related macular degeneration (iAMD).

**Methods:**

This cross-sectional, multicentric study enrolled 57 eyes of 43 iAMD patients. Drusen were subclassified as cuticular, soft, reticular pseudodrusen (RPD), or combined soft + RPD based on multimodal imaging. Dark adaptometry (AdaptDx; 20-minute test-time) assessed DA function through rod intercept time (RIT), last measured log sensitivity (LMLS; primary outcome) and area under the DA curve (AUDAC; secondary outcome). OCT volumes (30°x20° field) were analyzed using an AI-enhanced algorithm providing a quantification of chorioretinal layers and pigment epithelium detachments (PED) volumes. Multivariable tobit and linear regression analyzed associations between drusen phenotypes and DA outcomes.

**Results:**

Drusen phenotype distribution was: cuticular 13 eyes (22.8%), soft 13 eyes (22.8%), isolated RPD 21 eyes (36.8%), and combined soft + RPD 10 eyes (17.5%). Nearly all eyes reaching RIT within the 20-minute test ceiling (10 of 11 eyes) had cuticular drusen, which showed preserved rod function (LMLS: 2.9 ± 0.2 log-units; AUDAC: 10.3 ± 3.2 log-units·min; RIT: 14.9 ± 4.5 min) despite presenting the highest PED volumes (301 ± 152 nL). Conversely, isolated RPD demonstrated most severe dysfunction (LMLS: 1.7 ± 0.3; AUDAC: 23.7 ± 5.39). Soft drusen showed intermediate impairment (LMLS: 2.2 ± 0.2; AUDAC: 18.4 ± 2.4). Drusen phenotype was the most significant predictor of DA outcomes in both regression models (p < 0.001).

**Conclusion:**

Dark adaptometry reveals distinct patterns of DA impairment across drusen phenotypes, necessitating precise drusen classification when dark adaptometry serves as a functional endpoint in iAMD clinical trials. Future studies should employ extended test protocols (40-minute ceiling) to confirm these findings.

**Clinical trial number:**

Not applicable.

## Introduction

Dark adaptation (DA) has been proposed as a functional indicator of macular disease status progressively altered in patients affected with age-related macular degeneration (AMD) [1]. Given the strong plausibility of dark adaptation impairment linked to retinal aging [2], choriocapillaris (CC) dysfunction [3] and drusen biology [4], the measurement of DA has been suggested as a functional endpoint measure for interventional clinical trials in intermediate AMD (iAMD) [5]. 

According to current standardized measurements, dark adaptometry is a psychophysical test that measures the recovery of retinal sensitivity in a dark environment after the exposure to an intense photobleach [6]. The resulting DA curve consists of two principal phases: the initial short segment (S1) represents the exponential recovery of cone photoreceptors, reflecting the rapid kinetic response of the photopic visual system; the second segment (S2) represents the linear and slower rod-mediated recovery [1], where the dark-adapted scotopic threshold reaches up to 3 orders of magnitude lower than the photopic threshold (cone plateau) [7]. Direct measures of scotopic threshold include the rod-intercept-time (RIT), defined as the time required for rod sensitivity to recover by 3 log units [6], and the rod threshold, calculated by averaging stimulus luminances during the final minutes of testing [8]. Rod sensitivity (1/threshold in cd/m²) is inversely correlated with threshold: lower sensitivity values indicate elevated thresholds and impaired rod function. Additionally, the area under the DA curve (AUDAC) has been proposed as a mathematically derived curve metric that integrates the overall DA response [9], with demonstrated correlation with RIT [6]. 

Previous studies have disclosed the longitudinal variations of DA across the range of AMD severities, highlighting higher rates of RIT prolongation in eyes presenting with reticular pseudodrusen (RPD), also defined as subretinal drusenoid deposits (SDDs) [10]. Interestingly, SDDs show a peculiar zonal distribution, colocalized to areas of greater rod density [11], with strong implications for the prolongation of DA time. According to the ALSTAR2 trial (NCT04112667) investigators, measuring DA tested at 5° eccentricity from the fovea better differentiates between normal aging controls and iAMD: the 5° eccentric area, located at the interface between the inner and the outer ETDRS rings, is a vulnerable topographic area in which rod loss in aging is maximal and cones are less numerous [12]. In addition to SDDs and soft drusen, a supplemental drusen phenotype which frequently involves the 5°-area and presents a widespread spatial distribution, namely cuticular drusen [13], has been less studied in terms of impact on DA outcomes [14, 15].

We designed a cross-sectional study with the aim to investigate the influence of macular drusen phenotypes (i.e., cuticular drusen, soft drusen and reticular pseudodrusen) [16] on DA in patients diagnosed with iAMD. In this study, in addition to RIT and AUDAC outcomes, we evaluate the last measured log sensitivity (LMLS), representing the absolute rod sensitivity at completion of the selected test ceiling. Furthermore, given the growing interest in the relation between DA and the status of outer retinal layers [17], our secondary aim was to investigate the association of DA metrics with optical coherence tomography (OCT) outer retinal and choroidal layers volumetric features. This investigation was complemented by the analysis of the topographical distribution of macular drusen, facilitated by the combination of B-scan OCT and angiographic imaging techniques.

## Methods

### Study design and population

In this cross-sectional, exploratory, multicentric cohort study we collected the clinical records of consecutive treatment-naive patients diagnosed with iAMD established according to the Beckman criteria (drusen > 125 μm and/or pigmentary abnormalities associated with drusen) [18] and/or the presence of RPD/SDDs [19]. Eligibility criteria also included: (1) age ≥ 55 years; (2) visual acuity (Early Treatment Diabetic Retinopathy Study; ETDRS chart) > 50 letters; (3) absence of macular neovascularization (either exudative or non-exudative).

Recruitment of patients occurred between January 2023 and January 2024 from two academic retinal centers, specifically the University Eye Clinic DINOGMI at IRCCS San Martino Hospital (Genoa, Italy) and IRCSS Ca’ Granda Foundation Ospedale Maggiore Policlinico (Milan, Italy), in collaboration with Inselspital University Hospital (Bern, Switzerland). This non-interventional study was approved by the regional ethics committee of Liguria Region and was performed in agreement with the principles outlined in the Declaration of Helsinki for research involving human subjects.

Patients with the following conditions were excluded from the study: (1) any previous treatment for AMD; (2) any other chorioretinal or optic nerve disorder; (3) relevant optic media opacities that precluded the observation of the fundus and/or insufficient fixation to allow high-quality imaging and psychophysical tests; (4) insufficient pharmacological mydriasis, induced by to topical phenylephrine 100 mg/ml + tropicamide 5 mg/ml, measured ≤ 6.3 mm; (5) fixation error rate at DA examination ≥ 35%; (6) history of vitamin A deficiency or oral supplementation of retinyl palmitate > 10.000 Units per day, and history of acute or past liver diseases [20].

### Data collection and OCT analysis

Clinical data were retrieved by independent ophthalmologists (F.M. and M.N). In addition to demographic characteristics and laterality, the following clinical examinations were performed: best-corrected visual acuity (BCVA) testing using ETDRS chart at 4 m, dilated ophthalmoscopy, color fundus photography (DRI OCT Triton; Topcon Corporation, Tokyo, Japan), fundus autofluorescence (FAF), near-infrared reflectance (IR), Multicolor imaging, and OCT (Spectralis HRA + OCT; Heidelberg Engineering, Heidelberg, Germany). OCT volumes were obtained with a volumetric acquisition (30°x20° field, 97 sections, 1530 A-scans, automatic real time-function).

**Fig. 1:**
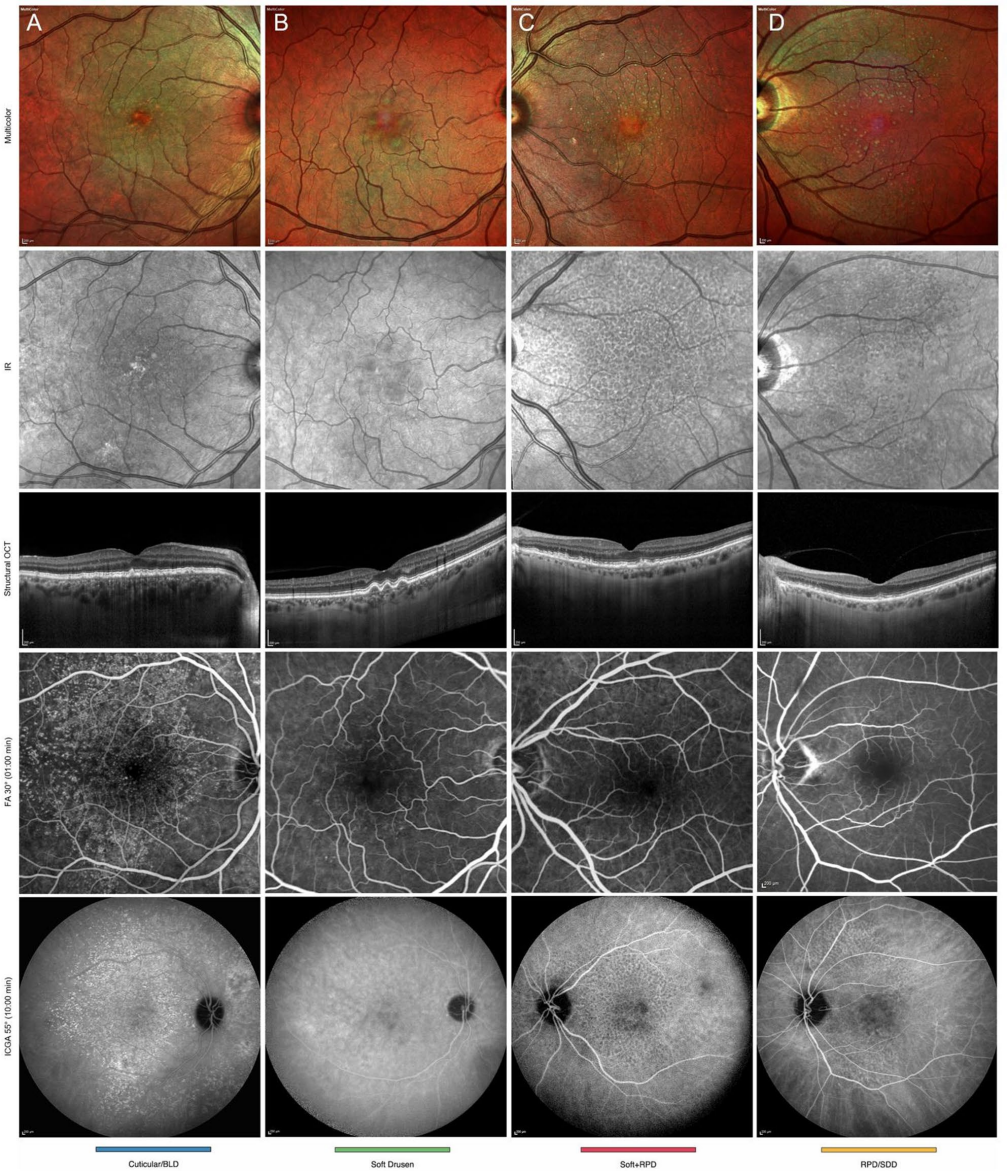
Example cases of the grading criteria adopted in the study Each row represents a different drusen phenotype (color-coded as in subsequent figures: blue = cuticular drusen;  green = soft drusen; red = soft + RPD; yellow = isolated RPD). From top to bottom, rows show: multicolor fundus photography, near-infrared reflectance (NIR), spectral-domain optical coherence tomography (OCT) B-scan, late-phase fluorescein angiography (FA), and indocyanine green angiography (ICGA). (*Column A*) Cuticular drusen: Multicolor imaging shows multiple small deposits. FA demonstrates discrete roundish hyperreflective lesions in a “stars-in-the-sky” pattern. ICGA reveals intensely hypercyanescent lesions. OCT shows widespread sub-RPE triangular elevations with saw-tooth pattern, hyporeflective cores, and signal hypertransmission at drusen apices. (*Column B*) Soft drusen: Multicolor imaging shows large confluent deposits. OCT reveals hyporeflective sub-RPE deposits with dome-shaped RPE elevations, minimally hyperfluorescent in FA, and with a subtle variation in the NIR grayscale tones. (*Column C*) Combination of soft drusen with RPD: NIR clearly reveals the presence of subretinal drusenoid deposits as hyporeflective lesions with a reticular pattern. ICGA shows hypocyanescent deposits in late frames. OCT demonstrates both sub-RPE soft drusen and subretinal drusenoid deposits. (*Column D*) RPD: NIR shows characteristic reticular pattern. OCT reveals subretinal hyperreflective deposits with circumferential parafoveal distribution, disrupting the ellipsoid band and protruding into outer retinal layers.

All multimodal images were subsequently graded by two independent graders (P.F. and M.N.) to confirm AMD classification and to classify the drusen type in analogy to characterization proposed by Spaide and Curcio [16]. Fluorescein angiography (FA) and Indocyanine Green Angiography (ICGA; Spectralis HRA + OCT) were performed as confirmatory imaging modalities in cases of suspected cuticular drusen. [13] Cuticular drusen were sub-classified into three patterns based on OCT features according to characterization proposed by Balaratnasingam et al. [13] RPD were sub-classified according to the characterization proposed by Zweifel et al. [21] The presence of RPD was considered either alone or in combination with other drusen phenotype. Example cases of the grading adopted in the study are shown in Fig. 1.

After anonymization and blinded to visual and functional DA results, OCT volumes were analyzed through the Discovery^®^ platform (Discovery OCT Fluid and Biomarker Detector, RetinAI AG, Switzerland) providing an automated quantification of retinal and choroidal layers thickness volumes. This software is based on a Convolutional Neural Network (CNN) architecture and was trained in a supervised manner, as demonstrated by the study published by Kurmann et al. [22]

In detail, the artificial intelligence-assisted measurement of retinal and choroidal thicknesses measures the retinal nerve fiber layer (RNFL), ganglion cell layer and inner plexiform layer (GCL + IPL), inner nuclear layer and outer plexiform layer (INL + OPL), outer nuclear layer (ONL), photoreceptor and RPE layers (PR + RPE) and choriocapillaris with choroidal stroma, and the overall retina thickness (RT). Volumetric analysis provides the pigment epithelium detachments (PED) volumes measured in nL, in analogy to previous studies [23]. When an error in automated thickness and/or volumes was present, manual editing was performed by a trained ophthalmologist (L.F.D.).

### DA protocol

Each eligible patient underwent DA examination using an adaptometer (AdaptDx, Maculogix, USA), a psychophysical test conducted in a dark room after pupil dilation (mydriasis > 6.3 mm). The patient was instructed to fixate on a central light (635 nm), and a near spherical correction was customized for each eye in the study (+ 3.00DS + spherical distance prescription confirmed by a trained optometrist). The protocol considered for DA testing was selected according to a critical review of previous literature on DA topics and related testing procedures [24]. Following intense photo bleach (82%) in a region 5° eccentric on the inferior visual meridian (≈ 1.50 mm superior to the fovea), with a circular diameter of 1.7° (≈ 0.51 mm) and a wavelength of 505 nm test spot. Starting with a stimulus of 5 cd/m^2^, consecutive threshold values (3down/1up staircase threshold estimate strategy) were tested, beginning 15 s after the bleaching flash, to a maximum of 3 log-units and a test time of 20 min (selected as test ceiling for feasibility in clinical practice [25]). The rod-intercept-time (RIT) was defined as the time in minutes required for a recovery of 3 log-units. In case of eligibility of both eyes, the DA examination was conducted at a maximum distance of 1 week in the fellow eye, starting from the eye with higher BCVA.

### Study endpoints

To separately analyze the static and dynamic components of DA, we employed two distinct response variables. The steady-state rod photoreceptor component was quantified through the LMLS as primary study endpoint which captures the final absolute sensitivity independently from DA kinetics. The dynamic component was assessed using the AUDAC approach, calculated via Simpson’s rule with an intercept of 3 log-units from minute 0 until the RIT time or a 20-minute cut-off time. While this measure integrates both rate-dependent and static components, it provides complementary information about the overall temporal pattern of sensitivity recovery [9]. 

### Statistical analysis

All statistical analyses were performed using R (Version 4.3.3). The Kruskal-Wallis Rank Sum test was utilized to assess differences among the different phenotypes of drusen concerning demographics, DA test and OCT parameters. We specifically focused on outer retinal and choroidal alterations as they represent the primary biological pathway implicated in iAMD pathogenesis [17]; this biological plausibility guided our a priori selection of covariates.

The joint effect of macular drusen subtypes (cuticular drusen, soft drusen, and reticular pseudodrusen), outer retinal (ONL and PR + RPE) and choroidal (CC + CS) layers volumes (1 mm^3^ increments), age (10 yrs increments) and BCVA (5 ETDRS letters increments) on LMLS was estimated using a multivariable tobit model (TM); this regression model specifically addresses the right-censored LMLS measurements of patients reaching the test ceiling of 3 log-units, providing estimates of the relationship between drusen phenotypes and absolute rod function.

The joint effect of the same covariates on AUDAC was estimated using a multivariable linear model (LM) as a complementary analysis of the dynamic component of dark adaptation. For readers interpretation purposes, preserved DA is indicated by higher LMLS values (positive τ regression coefficients in TM) and lower AUDAC values (negative β regression coefficients in LM).

Given that clinical measurements carried out on paired organs are in general positively correlated, to reduce the impact of inter-eye correlation on statistical inference, sampling variabilities of TM regression parameters and related 95% confidence limits (95%CL) were computed using a random-intercept model approach [26]; with analogous purpose, sampling variabilities of LM regression parameters and related 95%CL were computed using a generalized estimating equation approach (GEE) [27], which models the marginal mean response while accounting for within-subject correlation through an exchangeable working correlation matrix, with robust variance estimators providing valid inference for bilateral eye data. A two-sided probability value ≤ 0.05 was considered as statistically significant.

## Results

### Demographics and population

The study cohort comprised 57 eyes of 43 patients (63.2% female, mean age: 70.8 ± 8.1 yrs). All enrolled eyes displayed good visual acuity (78.6 ± 5.5 ETDRS letters) and 42/57 eyes (73.7%) were phakic. Drusen phenotypes were distributed as follows: cuticular drusen (22.8%), soft drusen (22.8%) RPD (36.8%), and a combination of soft drusen with RPD (17.5%). Among eyes with cuticular drusen, 6/13 eyes (46.3%) fit into the type 1 pattern, 7/13 (53.8%) type 2 pattern and 0/13 (0.0%) type 3 patterns; therefore, our results cannot elucidate if dark adaption is differently impaired in atypical cuticular drusen presenting with type 3 pattern [13].

### Zonal distribution

**Fig. 2:**
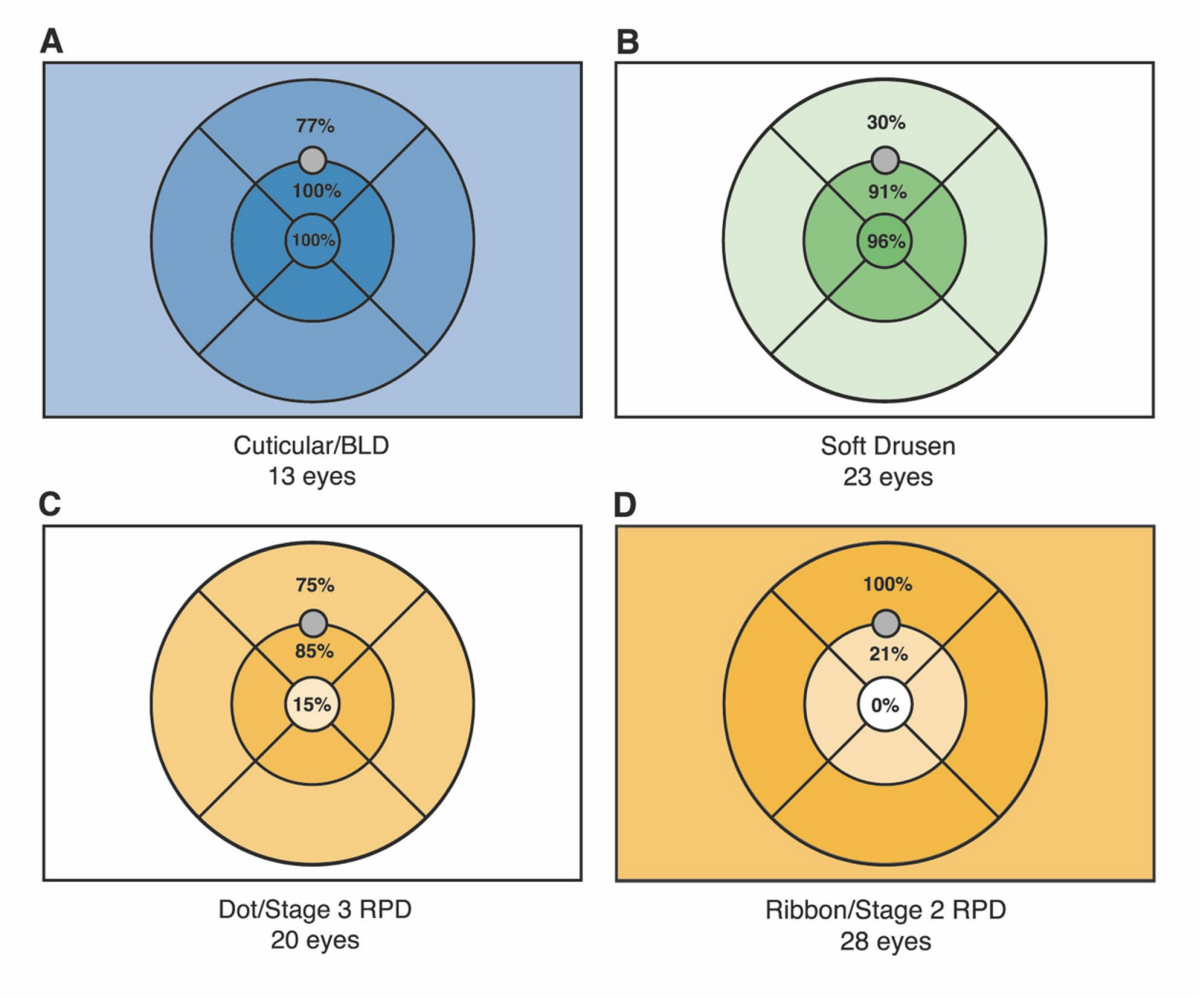
Zonal distribution of drusen within the OCT field and in relation to the ETDRS grid. The figure illustrates the preferential topographical distribution of the four drusen phenotypes analyzed: (**A**) Cuticular/BLD (blue) show widespread spatial distribution across all ETDRS fields, present in 100% of cases in the inner circle and inner ring, and in 76.9% in the outer ring. (**B**) Soft drusen (green) demonstrate predominantly central distribution, present in 91.3% of cases in the inner circle and progressively decreasing toward eccentric zones. (**C**-**D**) RPD (yellow) display a characteristic distribution at the interface between inner and outer rings: Dot/Stage-3 RPD are predominantly found in the inner ring (85.0%) and outer ring (75.0%), while Ribbon/Stage-2 RPD are less frequent in the inner ring (21.4%) and more prevalent in the outer ring. Note: Dark Adaptation 1.7°-diameter (≈0.51mm) region of interest (gray circle) lies 5° eccentrically on the inferior visual meridian (≈1.50mm superior to the fovea) at the interface between the inner and the outer ETDRS rings

Table 1; Fig. 2 show the preferential zonal distribution of drusen phenotypes in relation to the topographical region of interest, defined by the 30° x 20° OCT field and ETDRS concentric rings: (A) 13/57 eyes (22.8%) displayed the presence of cuticular/basal laminar drusen (BLD), with widespread spatial distribution of 13/13 (100.0%) in the inner circle and ring and 10/13 (76.9%) in the outer ring; (B) 23/57 eyes (40.3%; 13 soft only + 10 of concomitant soft and RPD group) displayed the presence of soft drusen, with a primary distribution in the inner circle in 21/23 eyes (91.3%) and some presence in the outer circle in 7/23 eyes (30.4%); (C) RPD display a characteristic distribution at the interface between inner and outer rings; when subclassified as Dot/Stage-3 and Ribbon/Stage-2, 20/57 eyes (35.1%) displayed the presence of Dot/Stage-3 RPD, with a predominant presence in the inner ring (17/20 eyes; 85.0%) followed by the outer ring (15/20 eyes; 75.0%); and (D) conversely, Ribbon/Stage-2 RPD (28/57 eyes; 49.1%) were less prevalent in the inner ring (6/28 eyes; 21.4%) and more frequent in the outer ring (28/28 eyes; 100.0%).

### DA test and OCT analysis

**Fig. 3:**
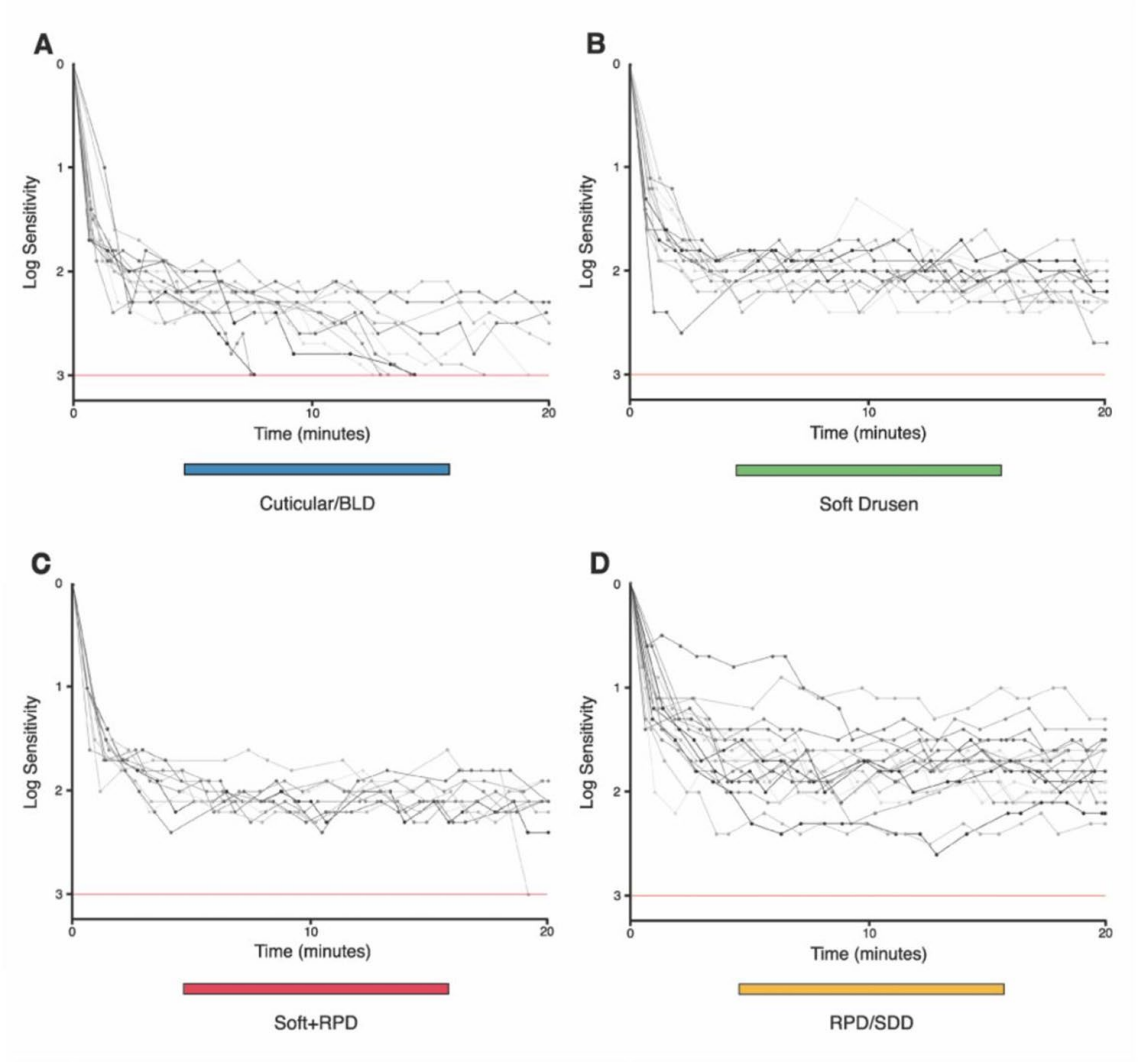
Rod-mediated dark adaptation curves in (**A**) cuticular drusen, (**B**) soft drusen, (**C**) combination of soft drusen with reticular pseudodrusen, and (**D**) isolated reticular pseudodrusen. Individual dark adaptation curves are shown clustered for drusen subtype. Each trace represents an individual eye's log sensitivity measurements over the 20-minute test time. Notably, only cuticular drusen eyes predominantly reached RIT within 20 minutes, despite having the highest drusen volumes. Note: The area under the dark adaptation curve (AUDAC) was calculated using Simpson’s rule with an intercept of 3.0 log-units (red horizontal line) from minute 0 until the rod-intercept-time (RIT) or a 20-minute cut-off time

In conducting the DA exam, the average percentage of fixation error rate was 0.14 ± 0.07. The mean pupil diameter was 7.5 ± 0.7 mm. In 46/57 eyes (80.7%) the RIT did not reach the 20-minute threshold i.e., projected RIT value > 20 min test ceiling. Among the 11 out of 57 eyes (19.3%) in which the RIT could be recorded within the testing time, 10/11 eyes displayed cuticular drusen (average intercept 14.9 ± 4.5 min) and 1/11 eye displayed soft + RPD (19.39 min). Dark adaptation curves stratified by drusen subtype are presented in Fig. 3.

Table 2 shows the distribution of clinical features and metric items in relation to drusen phenotypes. The AI-enhanced evaluation of OCT volumes field revealed additional information: cuticular drusen presented higher PED volumes (301 ± 152 nL; p-value < 0.001) and younger age (62.3 ± 7.7 yrs; p-value < 0.001). RPD displayed lower choroidal volume (6.80 ± 1.52 mm^3^; p-value < 0.001), and lower BCVA scores (77.2 ± 6.0 ETDRS letters; p-value < 0.001). Soft drusen showed intermediate features and a higher GCL + IPL volume (2.73 ± 0.22 mm^3^; p-value < 0.001). There were no significant differences in the remaining variables among the study participants (see Table 2).

Significant differences in AUDAC outcome were observed among macular drusen phenotypes (p-value < 0.001). For AUDAC, isolated RPD exhibited the highest values (23.7 ± 5.39 log-units⋅min; worst functional outcome) confirming a predominant rod dysfunction, followed by isolated soft drusen (18.4 ± 2.4 log-units⋅min), soft + RPD (18.5 ± 1.8 log-units⋅min) and cuticular drusen (10.3 ± 3.2 log-units⋅min). Correspondingly, LMLS analysis showed cuticular drusen with the highest threshold (2.9 ± 0.2 log-units; best functional outcome), followed by soft drusen (2.2 ± 0.2 log-units), soft + RPD (2.2 ± 0.3 log-units), and isolated RPD with the lowest values (1.7 ± 0.3 log-units; worst functional outcome).

**Fig. 4:**
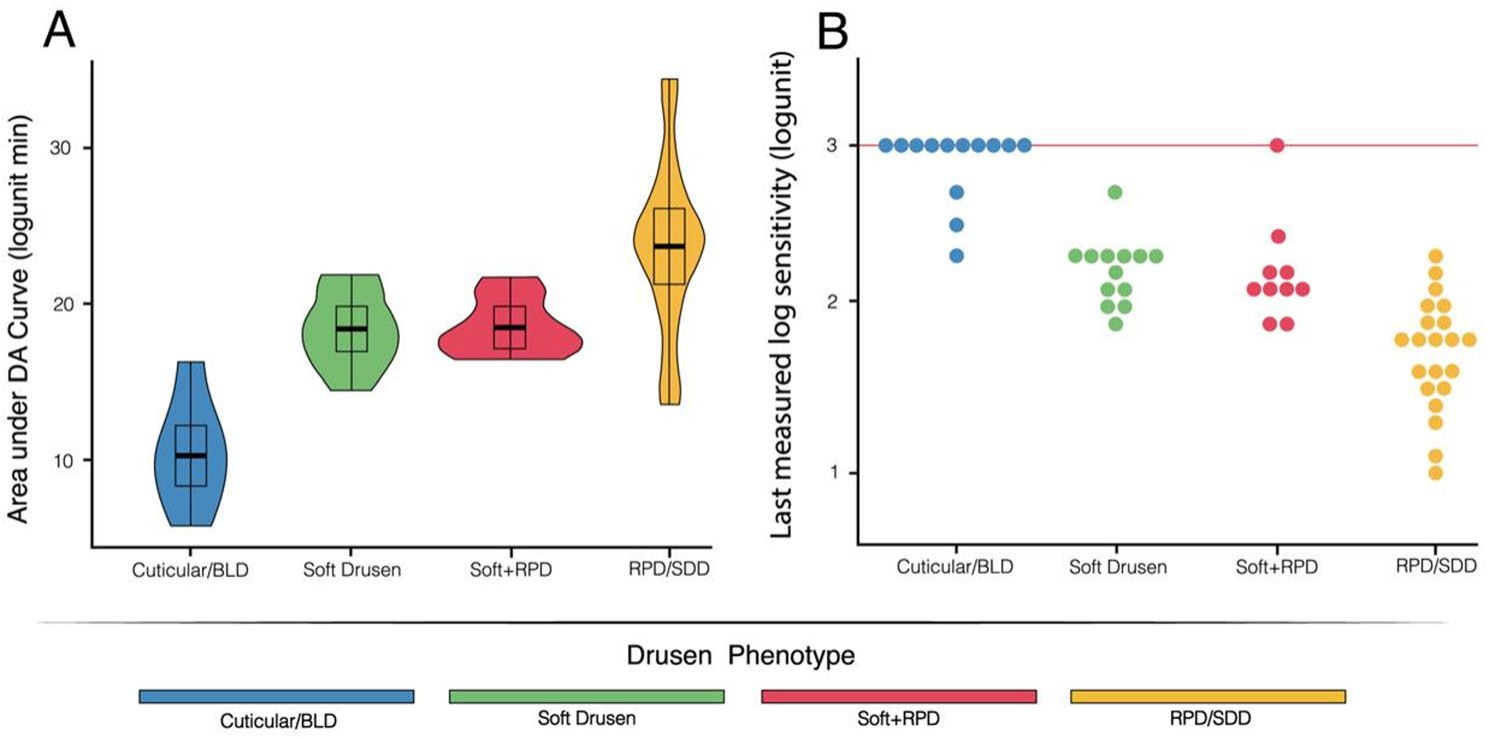
(**A**) Violin plots of the area under the dark adaptation curve (AUDAC) and (**B**) beeswarm plots of the last measured log sensitivity (LMLS) in relation to drusen phenotype. Legend – BLD: Basal Laminar Drusen; DA: Dark adaptometry; RPD: Reticular Pseudodrusen; SDD: Subretinal Drusenoid Deposits. Note: Among the eyes in which the rod intercept time could be recorded within the testing time, the LMLS value reached the right-censored scotopic threshold of 3.0 log-units (red horizontal line)

Violin plots and beeswarm plots displaying AUDAC and LMLS distributions in relation to drusen phenotype are displayed in Fig. 4.

### Tobit model

**Fig. 5:**
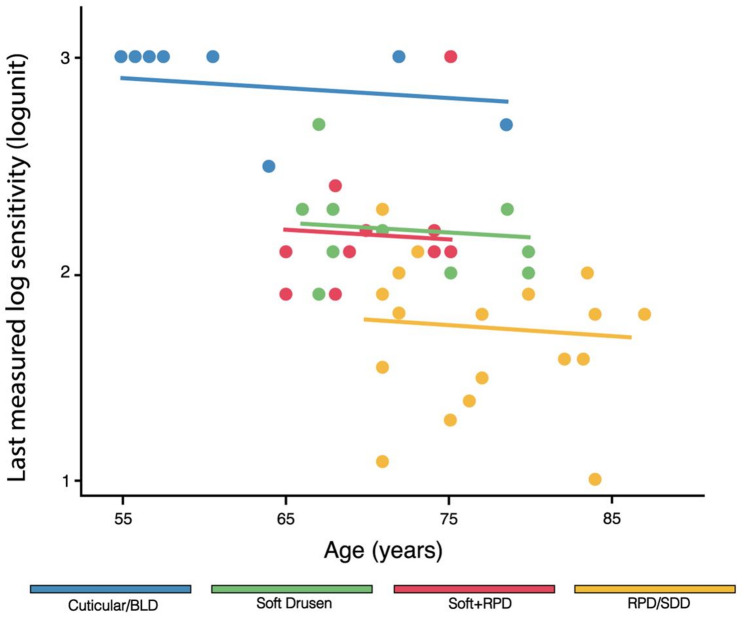
Scatter plot of the last measured log sensitivity (LMLS) trends as a function of age, according to the multivariable tobit model (TM) categories. Legend – BLD: Basal Laminar Drusen; RPD: Reticular Pseudodrusen; SDD: Subretinal Drusenoid Deposits. Note: While univariable analysis showed a significant association between age and dark adaptation (p-value < 0.001), this effect was attenuated in the multivariable TM. As visualized in the 65–75-year bracket, distinct macular drusen phenotypes stratify to specific LMLS thresholds, supporting an independent association between drusen phenotype and dark adaptation functional metrics

The multivariable TM with random-intercept implementation (Table 3) revealed a significant association between LMLS and drusen phenotype (p-value < 0.001): cuticular drusen were associated with better rod function (τ = 0.64, 95% CL = 0.34 / 0.93) despite presenting the highest PED volume. A positive association was also observed for better BCVA (linear trend per 5 ETDRS letters increase: τ = 0.01, 95% CL = 0.01 / 0.02, p-value = 0.043). While age and choroidal volume showed significant associations in univariable analysis (p-value < 0.001), these effects were attenuated in the multivariable model (age: τ = -0.06, 95% CL= -0.20 / 0.07, p-value = 0.366; CC + CS: τ = -0.01, 95% CL= -0.05 / 0.03, p-value = 0.646). Figure 5 shows LMLS trend as a function of age according to the TM categories. The presence of isolated RPD was associated with lower LMLS (τ = -0.41, 95% CL= -0.63 / -0.19, when referenced to the soft + RPD Group), indicating more severe rod dysfunction. The remaining OCT variables, including ONL (p-value = 0.121) and PR + RPE (p-value = 0.394), did not contribute significantly to LMLS among our cohort.

### Linear model

The multivariable LM (Table 4) corroborated the TM findings regarding drusen phenotype associations (p-value < 0.001) with consistent regression coefficients: cuticular drusen showed better adaptation (β = -6.18, 95% CL= -9.79 / -2.55), and isolated RPD exhibited worse outcomes (β = 4.00, 95% CL = 1.20 / 6.79, referenced to soft + RPD). BCVA remained significantly associated with AUDAC only in the univariable model, while ONL volume emerged as an additional predictor (β = -5.22, 95% CL= -9.34 / -1.09, p-value = 0.013). PR + RPE (p-value = 0.083), and CC + CS (p-value = 0.497) also showed no significant contributions.

**Table 1:** Zonal distribution of Drusen within the 30° x 20° OCT field and in relation to the ETDRS grid

Topographical region of interest	Drusen Phenotype (*n*, %)			
	Cuticular/BLD	Soft Drusen	RPD/SDD	
			Dot/Stage-3	Ribbon/Stage-2
Central Circle	13 (100.0%)	22 (95.6%)	3 (15.0%)	0 (0.0%)
Inner Ring	13 (100.0%)	21 (91.3%)	17 (85.0%)	6 (21.4%)
Outer Ring	10 (76.9%)	7 (30.4%)	15 (75.0%)	28 (100.0%)
Beyond ETDRS Grid*	8 (61.5%)	0 (0%)	0 (0%)	22 (70.9%)
Total	13 eyes	23 eyes	20 eyes	28 eyes

Legend – BLD: Basal Laminar Drusen; RPD: Reticular Pseudodrusen; SDD: Subretinal Drusenoid Deposits. *: Outside the Early Treatment Diabetic Retinopathy Study grid but within 30°x20° optical coherence tomography field

Note: Dark Adaptation 1.7°-diameter (≈0.51mm) region of interest lies 5° eccentrically on the inferior visual meridian (≈1.50mm superior to the fovea) at the interface between the inner and the outer ETDRS rings

**Table 2:** Distribution of categorical and metric items in relation to Drusen phenotypes among 57 eyes of 43 patients in the study

Drusen Phenotype	N (%)	Cuticular/BLD	Soft Drusen	Soft + RPD/ SDD	RPD/SDD	p-value
		13 (22.8)	13 (22.8)	10 (17.5)	21 (36.8)	
Demographics	Female Gender (%)	6 (60.0)	5 (54.5)	5 (83.3)	7 (46.7)	0.411#
	Age (years)	62.3 ± 7.7	71.9 ± 5.47	70.3 ± 3.8	77.5 ± 5.4	< 0.001*
Visual acuity and lens status	BCVA (ETDRS)	80.9 ± 4.9	79.2 ± 5.2	77.7 ± 5.4	77.2 ± 6.0	0.240
	Pseudophakia (%)	2 (15.3)	2 (15.3)	4 (40.0)	7 (33.3)	0.555#
OCT Analysis	Subfoveal ChT (µm)	259 ± 57	249 ± 66	162 ± 53	141 ± 54	< 0.001*
	RNFL (mm^3^)	2.48 ± 0.23	2.44 ± 0.41	2.53 ± 0.35	2.54 ± 0.45	0.870
	GCL + IPL (mm^3^)	3.03 ± 0.16	2.73 ± 0.22	2.78 ± 0.19	2.75 ± 0.25	< 0.001*
	INL + OPL (mm^3^)	2.41 ± 0.10	2.33 ± 0.15	2.35 ± 0.18	2.32 ± 0.23	0.537
	ONL (mm^3^)	3.50 ± 0.20	3.52 ± 0.33	3.16 ± 0.25	3.42 ± 0.45	0.537
	PR + RPE (mm^3^)	3.00 ± 0.18	2.93 ± 0.17	3.04 ± 0.23	2.98 ± 0.33	0.767
	CC + CS (mm^3^)	11.6 ± 1.69	9.50 ± 1.94	7.75 ± 1.77	6.80 ± 1.52	< 0.001*
	PEDV (nL)	301 ± 152	110 ± 142	94 ± 57	19 ± 23	< 0.001*
Dark Adaptometry Metrics	Fixation Error (%)	11.8 ± 5.6	14.6 ± 9.7	17.5 ± 9.6	14.5 ± 5.6	0.451
	Test Time (min)	14.6 ± 2.8	15.6 ± 1.0	16.3 ± 1.2	15.6 ± 1.9	0.206
	Pupil Size (mm)	7.7 ± 0.5	7.5 ± 0.6	7.9 ± 0.7	7.2 ± 0.7	0.128
	AUDAC (log-units⋅min)	10.3 ± 3.2	18.4 ± 2.4	18.5 ± 1.8	23.7 ± 5.39	< 0.001*
	LMLS (log-units)	2.9 ± 0.2	2.2 ± 0.2	2.2 ± 0.3	1.7 ± 0.3	< 0.001*

Legend – AUDAC: Area under the dark adaptation curve; BLD: Basal Laminar Drusen; ETDRS: Early Treatment Diabetic Retinopathy Study letters; mm: millimeter; N: number; LMLS: last measured log sensitivity; OCT: Optical Coherence Tomography; RPD: Reticular Pseudodrusen; SDD: Subretinal Drusenoid Deposits; p-value: predictivity value according to the Kruksal-Wallis Rank Sum test. *: statistically significant results. #: p-value according to the chi-squared test

**Table 3:** Multivariable Tobit model (TM) of last measured log sensitivity (LMLS) based on Drusen phenotypes, OCT volumetric analysis, age and BCVA

Tobit Model with random-intercept implementation							
Variable		Univariable			Multivariable		
		τ	95%CL	P-value	τ	95%CL	P-value
Drusen phenotype	Cuticular/BLD	0.89	0.57/1.22	< 0.001*	0.64	0.34/0.93	< 0.001*
	Soft Drusen	-0.01	-0.29/0.29		0.02	-0.21/0.25	
	RPD/SDD	-0.49	-0.77/-0.22		-0.41	-0.63/-0.19	
	Soft + RPD/SDD	0.00	(Ref.)		0.00	(Ref.)	
Volumetric OCT Analysis *Linear trend per* *1 mm*^*3*^ *increase*	ONL	-0.13	-0.50/0.24	0.491	0.27	-0.07/0.61	0.121
	PR + RPE	-0.17	-0.64/0.30	0.471	-0.20	-0.67/0.27	0.394
	CC + CS	0.14	0.08/0.20	< 0.001*	-0.01	-0.05/0.03	0.646
Age *Linear trend per 10 yrs increase*		-0.59	-0.77/-0.41	< 0.001*	-0.06	-0.20/0.07	0.366
BCVA *Linear trend per 5 ETDRS letters increase*		0.03	0.01/0.05	0.024*	0.01	0.00/0.02	0.043*
Constant		-	-	-	2.18	2.01/2.35	-

*Legend* - τ: tobit regression coefficient; 95%CL: 95% confidence limits for τ; BLD: Basal Laminar Drusen; CC + CS: choriocapillaris with choroidal stroma; ONL: outer nuclear layer; PR + RPE: photoreceptor and retinal pigment epithelium layers; RPD: reticular pseudodrusen; SDD: subretinal drusenoid deposits; P-value: probability level associated with the Wald test; *: statistically significant results

Note - Constant: Fitted LMLS values (log-units) when “Drusen Phenotype” = “Ref.” and all other predictors are equal to their mean levels

**Table 4:** Multivariable linear model (LM) of area under DA curve (AUDAC) based on Drusen phenotypes, OCT volumetric analysis, age and BCVA

Linear Model with GEE implementation											
		Univariable					Multivariable				
Variable		β		95%CL		P-value	β		95%CL		P-value
Drusen phenotype	Cuticular/BLD	-8.19	-11.2/-5.23		< 0.001*		-6.18	-9.79/-2.55		< 0.001*	
	Soft Drusen	-0.22	-3.20/2.75				1.06	-1.94/4.05			
	RPD/SDD	5.25	2.55/7.95				4.00	1.20/6.79			
	Soft + RPD/SDD	0.00	(Ref.)				0.00	(Ref.)			
Volumetric OCT Analysis *Linear trend per* *1 mm*^*3*^ *increase*	ONL	-1.83	-6.21/2.54		0.412		-5.22	-9.34/-1.09		0.013*	
	PR + RPE	0.71	-4.99/6.47		0.800		6.03	0.39/11.6		0.083	
	CC + CS	-1.62	-2.12/-1.12		< 0.001*		-0.20	-0.75/0.36		0.497	
Age *Linear trend per 10 yrs increase*		5.48		3.92/7.03		< 0.001*	0.54		-1.18/2.25		0.539
BCVA *Linear trend per 5 ETDRS letters increase*		-0.47		-0.70/-0.25		< 0.001*	-0.30		-0.47/-0.12		< 0.001*
Constant		-		-		-	18.30		16.0/20.6		-

*Legend* - β: linear regression coefficient; 95%CL: 95% confidence limits for β; BLD: Basal Laminar Drusen; CC + CS: choriocapillaris with choroidal stroma; GEE: generalized estimating equation; ONL: outer nuclear layer; PR + RPE: photoreceptor and retinal pigment epithelium layers; RPD: reticular pseudodrusen; SDD: subretinal drusenoid deposits; P-value: probability level associated with the Wald test; *: statistically significant results

Note - Constant: Fitted AUDAC values (log-units⋅min) when “Drusen Phenotype” = “Ref.” and all other predictors are equal to their mean levels

## Discussion

Dark adaptometry testing serves as a thermometer of photoreceptors functional impairment in patients affected with AMD. Our findings point to a continuous spectrum of DA impairment among iAMD patients. Using LMLS as our primary outcome through the TM, we found significant associations between macular drusen phenotypes (p-value < 0.001) and BCVA (p-value = 0.043) with DA functional metrics. These findings were corroborated by our complementary analysis of the AUDAC through the LM, which revealed an additional significant association with ONL volume (p-value = 0.013). The integration of comprehensive multimodal imaging and DA testing, enhanced by AI-assisted OCT analysis, allowed us to investigate the relationship between specific drusen phenotypes and photoreceptor’s function.

We speculate that the differential impairment of DA among drusen phenotypes may be explained by the different pathways of rod involvement in the biology of drusen, and by the regional differences in rod/cone density ratio, which have been estimated as 0.27 in the ETDRS grid central circle, 3.17 in the inner ring, and 10.12 in the outer ring [4].

Among the study eyes, 13/57 (22.8%) displayed the presence of cuticular drusen; this drusen subtype exerted a minor impact on DA, despite presenting the highest PED volumes; this aspect is of particular clinical interest since in previous prospective studies evaluating the longitudinal changes of DA [5, 9, 10, 28], the prevalence of cuticular drusen among the study population has not been explicitly reported. Cuticular drusen, also known as BLD, were first introduced by Gass in 1985 [29] as a phenotype exhibiting fluorescence during the early arteriovenous FA phase, with numerous puncta of staining known as the “stars-in-the-sky” pattern [29]. Other distinguishing characteristics include shallow “sawtooth” RPE elevations on B-scan OCT [15], pinstripe hypertransmission defects and dot hypoautofluorescence [13]. Interestingly, while contrast sensitivity impairment, dyschromatopsia [30], and minor impact on visual sensitivity [31] have been associated with cuticular drusen, subjective night blindness or rod dysfunction have not been reported: a study by Pfau et al. has supported the concept of predominant foveal cone dysfunction in cuticular drusen through the use of mesopic fundus-controlled perimetry [14]. Genetic studies have linked mutations in fibulin-3 (extracellular matrix protein located in the Bruch Membrane), fibulin-5 (FBLIN5; endogenous inhibitor of angiogenesis), complement factor H, and C3 convertase stabilizing antibody 3 nephritic factor (membranoproliferative glomerulonephritis type II) genes to early-onset cuticular drusen [32]; interestingly these genes do not directly interfere with rod photoreceptors activity. From a clinical standpoint, one of the most frequently described complications of cuticular drusen (up to 24.2% [13]) is acquired vitelliform lesions (AVLs) [29], which colocalize to the subfoveal cone-rich area, and may further suggest the preferential impairment of cone photoreceptors occurring in this drusen subtype.

Lying on the opposite pole of the DA impairment spectrum, the presence of RPD has been independently associated with slowed rod kinetics and impaired sensitivity in the paracentral macula; [5, 10, 33, 34, 35] our results confirm this finding with more compromised DA indices in the isolated RPD group. RPD preferentially locate in the superior perifovea, an area where there is a high density of rod photoreceptors [11]; moreover, rod photoreceptors morphology is altered at RPD location in the subretinal space above the RPE [21]. Additionally, RPD are found in non-AMD retinopathy (i.e., pseudoxantoma elasticum) in which DA is impaired [36]. 

A recent study by Kumar et al. [37] has described how local rod-mediated visual function is associated with the overall presence but not local extent of RPD, indicating diffuse pathogenic changes occurring in eyes with RPD. This notion is supported by histologic studies, in which the ONL is thinned in the areas immediately juxtaposed to the RPD [19, 38] The presence of RPD has been linked to a reduction of the ONL volume in patients with reticular pseudodrusen as compared to soft drusen, irrespective of subfield location [39]. AI-based measurements of ONL thickness have linked ONL thinning with marked loss of retinal function [40]. Although ONL volumes did not differ significantly in RPD group in our descriptive analysis (Table 2), the multivariable LM (Table 4) confirmed that ONL preservation independently contributes to better dark adaptation after adjusting for drusen phenotype.

In contrast to panretinal cuticular drusen, and to perifoveal RPD, soft drusen preferentially localize in the central foveal region and display a lipid-rich composition localized underneath the RPE [41]. The deposition of membranous debris and esterified cholesterol composing dome-shaped soft drusen may be driven by aging and increasing metabolic demand in the fovea region [42]. In our cohort, the isolated soft drusen group (13/57 eyes; 22.8%) displayed intermediate DA indices. This finding is consistent with the report by Pollreisz et al. [43], which correlated the abundance of soft drusen in the central subfield (> 20-fold higher in central ETDRS subfields than the outer rings) to the impairment of subfoveal lipid metabolism linked to cone biology.

The TM and LM analyses additionally revealed a significant negative correlation between improved DA indices and better BCVA scores. Since BCVA is a photopic test which evaluates the functionality of the central cones-dominated fovea (≈ 0.8 mm or 2.75°), when there is a reduction in BCVA measure, it is likely that the parafoveal rod damage (≈ 3.5–10° from fixation) has already outnumbered the subfoveal cone loss, resulting in decreased scotopic LMLS measurements at 5° eccentricity. Therefore, this finding is consistent with the natural history of AMD, in which rod dysfunction precedes cone dysfunction [44]. 

Dark adapted kinetics have been used as secondary outcome measures in several gene therapy trials [45]. There are three registered interventional AMD trials on clinicaltrials.gov utilizing dark adaptation techniques as an outcome measure (NCT05932069, NCT02848313, NCT06237127). In order to establish the utility of a test as an outcome measure, its reliability must first be assessed. A recent systematic review of the AdaptDx reported variable sensitivity and specificity rates for different types of AMD depending on the test conditions, with significant numbers of individuals unable to complete testing. The test-retest repeatability was also poor [24]. Delayed dark adaptation does precede the development of AMD, however long-term studies needs to be a minimum of three years in order to show these changes [46]. AMD occurs in an elderly population, who are also prone to lens opacities. The AdaptDx measurements can be affected by lens status [24]. Dark adaptometry may be a very useful technique, however, further investigation is required to better understand the specific subtypes of AMD in which it will be most useful [33]. 

Limitations of this study should be acknowledged. With regard to dark adaptation testing, the selected test ceiling for the study was relatively short (20 min) to enhance protocol clinical feasibility and reduce patient and examiner burden, compared to alternative research protocols such as ALSTAR2 trial setting (40 min). We acknowledge that longer test durations would be preferable, as only 11/57 eyes (19.3%) reached RIT within our 20-minute protocol, with 10/11 being cuticular drusen cases. Despite this constraint, our analytical approach successfully achieved phenotypic stratification across drusen types. Previous studies have also demonstrated AUDAC offers comparable diagnostic sensitivity in eyes where RIT could not be recorded within the testing time [9]. Consequently, the AUDAC approach combined with LMLS, which represents a direct biological parameter approach enabled us to establish phenotypic stratification despite the shorter test duration.

Additionally, BCVA testing using ETDRS may not be the optimal test to evaluate central visual function, as it only shows alterations in the most advanced stages of the disease. A possible alternative marker may be low-luminance-VA (LLVA), a method which involves assessing VA on a logMAR chart using an additional 2.0 Log neutral density filter in front of the tested eye [47]. LLVA is a mesopic measure (1.6 cd/m^2^ or less) which evaluates the function of a slightly larger area of macular sensitivity than standard VA, and detects central visual function changes in the presence of preserved standard VA [48]. 

The sample size for the study was relatively small, however previous studies on the DA topic were based on pooled data of increasing AMD severities, ranging from normal aging changes to late AMD and GA; conversely, our findings focused on the same stage of the disease (iAMD). This could explain why age did not contribute in noteworthy manner to the DA outcomes recorded in this homogeneous cohort of iAMD patients. While univariable analysis showed a significant association between age and dark adaptation (p-value < 0.001), this effect was attenuated in the multivariable models.

Lastly, the dynamic dark adaptometer used in this study relies on few-viewing stimulus presentation; new fundus-controlled devices are currently under development [49] and will provide a more suitable approach to assess small, localized regions-of-interest in patients with unstable fixation.

In conclusion, in treatment-naïve patients with iAMD, macular drusen phenotypes impact DA, with reticular drusen and cuticular drusen respectively exhibiting the lowest and highest values of LMLS. Dark adaptometry is a promising outcome measure for clinical trials and provides valuable insights into assessing functional aspects of iAMD. Nonetheless, when DA is selected as a response variable in clinical studies, it should be integrated with precise drusen phenotyping. This combined structural and functional approach allows better understanding of the selective DA pathway impairment across different phenotypes of macular drusen. Extended test protocols (40-minute test ceiling) in future studies will be valuable to capture complete rod recovery kinetic, particularly in severely impaired iAMD phenotypes.

## Data Availability

The datasets used during the current study are available from the corresponding author upon reasonable request.

## Abbreviations

AMD: Age-related macular degeneration
AUDAC: Area under dark adaptation curve
BCVA: Best-corrected visual acuity
BLD: Basal laminar drusen
CC + CS: Choriocapillaris with choroidal stroma
DA: Dark adaptation
ETDRS: Early Treatment Diabetic Retinopathy Study
FA: Fluorescein angiography
FAF: Fundus autofluorescence
GCL + IPL: Ganglion cell layer and inner plexiform layer
GEE: Generalized estimating equation
iAMD: Intermediate age-related macular degeneration
ICGA: Indocyanine green angiography
INL + OPL: Inner nuclear layer and outer plexiform layer
IR: Infrared reflectance
LM: Linear model
LMLS: Last measured log sensitivity
LLVA: Low-luminance visual acuity
OCT: Optical coherence tomography
ONL: Outer nuclear layer
PED: Pigment epithelium detachment
PR + RPE: Photoreceptor and retinal pigment epithelium
RIT: Rod-intercept time
RNFL: Retinal nerve fiber layer
RPD: Reticular pseudodrusen
SDD: Subretinal drusenoid deposits
TM: Tobit model

## Declarations

### Meeting presentation

This study was presented at the International Retinal Imaging Society (IntRIS) Symposium 2024, Los Angeles, CA.
